# Circular RNAs as a potential source of neoepitopes in cancer

**DOI:** 10.3389/fonc.2023.1098523

**Published:** 2023-04-12

**Authors:** Jiaqi Xia, Shuai Li, Baorui Ren, Pengxia Zhang

**Affiliations:** Key laboratory of Microecology-Immune Regulatory Network and Related Diseases, School of Basic Medicine, Jiamusi University, Jiamusi, Heilongjiang, China

**Keywords:** circular RNAs, human leukocyte antigen, neoepitope, immunotherapy, cancer

## Abstract

Neoepitopes have attracted much attention as targets for immunotherapy against cancer. Therefore, efficient neoepitope screening technology is an essential step in the development of personalized vaccines. Circular RNAs (circRNAs) are generated by back-splicing and have a single-stranded continuous circular structure. So far, various circRNAs have been poorly characterized, though new evidence suggests that a few translated circRNAs may play a role in cancer. In the present study, circRNA was used as a source of neoepitope, a novel strategy as circRNA-derived neoepitopes have never been previously explored. The present study reports CIRC_neo (circRNA-derived neoepitope prediction pipeline), which is a comprehensive and automated bioinformatic pipeline for the prediction of circRNA-derived neoepitopes from RNA sequencing data. The computational prediction from sequencing data requires complex computational workflows to identify circRNAs, derive the resulting peptides, infer the types of human leukocyte antigens (HLA I and HLA II) in patients, and predict the neoepitopes binding to these antigens. The present study proposes a novel source of neoepitopes. The study focused on cancer-specific circRNAs, which have greatly expanded the source pool for neoepitope discovery. The statistical analysis of different features of circRNA-derived neoepitopes revealed that circRNAs could produce long proteins or truncated proteins. Because the peptides were completely foreign to the human body, they could be highly immunogenic. Importantly, circRNA-derived neoepitopes capable of binding to HLA were discovered. In the current study, circRNAs were systematically analyzed, revealing potential targets and novel research clues for cancer diagnosis, treatment, and prospective personalized vaccine research.

## Introduction

The clinical objective of immunotherapy is to utilize the natural immune system of the human body for targeting and destroying cancer cells, thereby improving the overall survival rate of cancer patients (1). Therefore, identifying therapeutic targets for cancer-specific cytotoxicity is critical because it will provide direction for an individualized therapeutic regimen. Cancer immunotherapy based on neoepitopes recognized by T cells is critical to the success of anticancer treatment regimens (2). Neoepitopes are cancer-specific peptides that can stimulate the immune system more effectively in the human body. Neoepitope vaccines have shown remarkable efficacy in various types of cancer and are revolutionizing cancer treatment (3, 4). Therefore, accurate and rapid identification of neoepitopes plays a key role in cancer immunotherapy.

Recent studies have reported that transcripts harbor frameshift mutations, while aberrant splicing patterns may produce antigenic peptides (5–8). Therefore, tumor non-mutated source neoepitopes could represent an alternative to somatic mutation-derived neoepitopes as targets in cancer immunotherapy. Intron retention (IR) and alternative splicing (AS) may produce a novel class of cancer-specific neoepitopes, as reported in certain previous studies (7, 8). Neoepitopes are derived from genomic mutations in tumor cells, protein variations might also result from abnormal RNA processing. Abnormal RNA transcripts are overexpressed in tumor cells, and conditions exist for ribosome translation which can produce abnormal peptides. Peptides derived from abnormal RNA transcripts—peptides—may be presented on human leukocyte antigen (HLA) and serve as a source for neoepitopes. Circular RNAs with coding potential represent another potential source of neoepitopes, although these have not been explored to date.

CircRNAs are single-stranded continuous circular structures without polyadenylated tails, produced through back-splicing, and may be regulated by certain splicing factors (9). CircRNAs are suggested to regulate various cellular processes *via* different mechanisms, including interactions with microRNAs and RNA-binding proteins (RBP), consequently having important biological functions in eukaryotes (10). The structural stability, tissue specificity, and relatively high expression levels of circRNAs in exosomes, blood, and plasma (11) have led circRNAs to be recognized as promising biomarkers for disease diagnosis and prognosis. The consistent findings regarding the functions of circRNAs, their participation in various biological processes, and their carcinogenic potential have rendered circRNAs attractive molecules for general as well as cancer research (12). The development of non-polyadenylated RNA-seq and circRNA-seq, as well as bioinformatics-based analysis, has allowed the detection of thousands of circRNAs. Several circRNA detection and quantification software programs are currently available (13).

CircRNAs were initially considered a group of endogenous non-coding RNAs (ncRNAs) (14, 15). Similar to most ncRNAs, circRNAs were previously assumed to be untranslatable due to a lack of evidence for the presence of open reading frames (ORFs). Recent research, however, has revealed a comprehensive analysis of the coding potential of circRNAs (10). CircRNAs have been found to encode proteins in several studies (16–19). Certain circRNAs have even been shown to be translated *in vivo via* various internal ribosome entry sites (IRESs) (17, 20). Because the ORFs in circ-RNAs have a covalently closed structure, they can be translated multiple times, increasing the transcriptome and proteome complexity (17). Certain proteins encoded by circRNAs have been reported to play a key role in regulating the growth of cancer cells, with accumulating evidence suggesting that circRNAs are involved in tumorigenesis (12, 21, 22). Functionally, the translation of circRNAs may contribute to proteomic diversity by causing inframe internal deletions or frameshifts in the encoded proteins. An increasing number of studies are reporting the ubiquitous nature of circRNAs translation and that the long half-life of circRNAs leads to the accumulation of translated protein sequences. In addition, circRNAs are highly expressed in cancer. Therefore, circRNAs are expected to serve as an excellent source of neoepitopes.

In the present study, the first automated high-throughput data analysis pipeline to detect circRNAs, predict its protein-coding potential and identify circRNA-derived peptides binding to HLA was developed and designated as CIRC_neo. RNA-seq data were used to identify circRNAs, which were then translated into protein sequences. Subsequently, the binding of the generated peptides to HLA was predicted. The identified binding peptides could serve as sources of neoepitopes. A strict screening process was adopted in the identification process to ensure that only those peptides that presented HLA and exhibited adequate expression levels were selected, as these were most likely to produce the required immune response when these were used in the follow-up studies.

To construct a catalog of cancer-specific circRNAs, CIRC_neo was applied to identify thousands of distinct circRNAs from the ribosomal RNA-depleted total RNA-seq or circRNA-seq data of human cancer tissues and normal tissues. Following that, cancer-specific neoepitopes were systematically identified and characterized using this catalog. Furthermore, the relationship between these neoepitopes and immunity and clinical practice was investigated. The current study suggested that circRNAs are a new source of cancer neoepitopes.

## Materials and methods

### Data source and preprocessing

**Figure 1:**
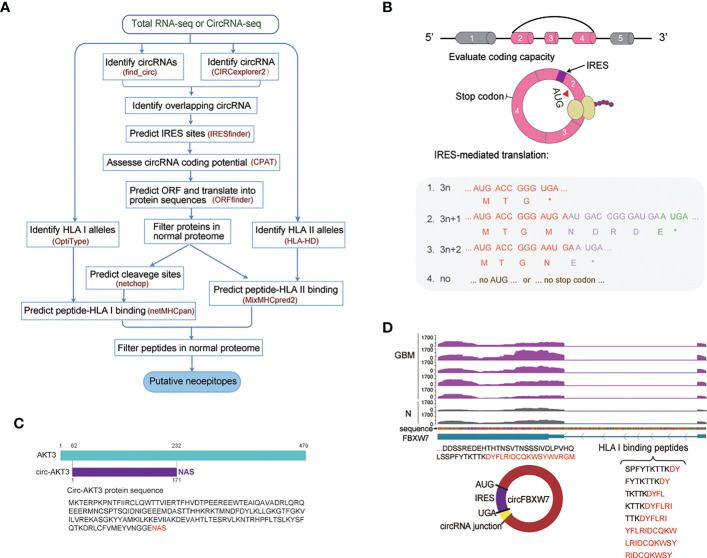
Computationally predicted neoepitopes detected in cancer patients. **(A)** Overview of the identification of putative HLA-specific neoepitopes using the developed CIRC-neo pipeline. **(B)** Schematic diagram of the circRNA production and translation process. Each box in the splice graph represents an exon, while each arc represents a splice. **(C)** Illustration of the AKT3 sequence and the circ-AKT3 protein sequence. **(D)** The upper panel depicts the track of FBXW7 in GBM and normal tissues. The lower left panel depicts the sequences of the putative protein and the putative open reading frame (ORF) in circFBXW7. The lower right panel depicts the peptides that could bind to HLA class I.

The published datasets of glioblastoma (GBM), bladder cancer (BC), and chronic lymphocytic leukemia (CLL) were used for collecting the data. The GBM circ-RNA-seq FASTQ files associated with Y. Liu et al. were downloaded from NCBI SRA: PRJNA525736 (23). The GBM dataset included data from 12 pairs of human GBM tissues and paired normal tissues. The ribosomal RNA-depleted total RNA from three pairs of BC tissues and paired normal bladder tissues were downloaded from NCBI GSE97239 (24). The third dataset included total RNA sequencing FASTQ files from 13 patients with CLL and 2 normal tissues obtained from NCBI GSE111793 (25). Quality control and preprocessing using FastQC. RNA reads were preprocessed to remove adaptor sequences and then mapped with STAR [26] to the hg19 reference genome. Then, gene expression quantization (FPKM) and Kyoto Encyclopedia of Genes and Genomes (KEGG) enrichment analyses were performed. Subsequent analyses were conducted, as shown in **Figure 1A**.

### Identification and annotation of circRNAs

Raw FASTQ reads aligned to the reference rRNA, and tRNA genome using bowtie2 were discarded. Next, STAR (26) was employed to map the unmapped RNA reads to human reference genomes. The anchor reads that aligned in the reverse orientation (head-to-tail) indicated circRNA splicing and were, therefore, subjected to analysis using find_circ (27, 28) to identify the circRNAs. A candidate circRNA was called if it was supported by a minimum of two unique back-spliced junction (BSJ) reads from at least one sample (≥2 BSJ reads for each candidate detected using any pipeline). GBM, BC, and CLL were used to identify circRNAs, which were analyzed separately.

### CircRNAs translation

CircRNAs require two basic elements for translation: IRES and ORF. IRESfinder (29) is a computational program that performs a comprehensive IRES search in a cell. To accurately and quantitatively evaluate the coding potential of circRNAs, CPAT (30) and CPC2 (31) were employed to assess the coding potential of RNA sequences. In the current study, the nucleotide sequence of the RNA was used as the input, and CPAT was used as a pre-built logistic model for measuring protein-coding likelihood, producing an output probability of P (0–1) with a selected probability threshold of ≥ 0.364. The RNA sequence identified as coding was presented in the CPC2 result file, and the intersection with the CPAT prediction was subjected to downstream analysis. All possible ORFs were identified using the ORFfinder tool.

### HLA genotyping

The input data used to detect the human leukocyte antigen (HLA) allele in the present study comprised the cleaned FASTQ files. Normal tissue RNA sequencing and cancer tissue RNA sequencing were performed for the 4-digit HLA class I and II determination. The OptiType version 1.3.1 (32) and HLA-HD version 1.2.0 (33) programs were used to detect the class-I and class-II HLA genotyping for each sample. OptiType, which presents improved accuracy compared to the other HLA determination tools (34), was used with the default setting. HLA class II determination was performed using HLA-HD. The HLA-HD tool reported the HLA types with 4-digit verification accuracy.

### HLA and peptide binding prediction

NetMHCpan4.0 is commonly used to predict patient-specific HLA class I binding peptides (35) as it considers algorithm benchmarks in large-scale evaluations and due to its availability across HLA alleles. In addition to peptides, the patient’s HLA genotype should be used as input for NetMHCpan4.0. When comparing binding among multiple HLAs, the percentage rank deviates less than the binding affinity metric, so it was used for neoepitope filtering. Each peptide with a predicted binding rank of ≤ 2.0% for at least one HLA class I allele could be designated neoepitope in all patients. In the HLA class II binding prediction using MixMHCpred2 (36), the 12–25-mers containing the circRNA-derived peptide for binding to the patient-specific HLA II were evaluated. The predicted rank percentage threshold that was applied for HLA class I was applied for nominating the HLA class-II-binding neoepitopes.

### Cancer epitope information is available in the IEDB

T-cell epitopes for cancer were identified by searching the IEDB (The Immune Epitope Database; http://www.iedb.org/) on June 22, 2022. Queries were performed broadly for Homo sapiens (ID:9606, human), Epitope Structure (Linear Sequence), Host: Homo sapiens (human), Disease Data: cancer (ID:162), and selecting positive assays in the contexts of T-cell and MHC ligands. The characteristics of each unique epitope (i.e., species, protein of provenance, positive assay type, MHC restriction) were tabulated. The IEDB had 264668 curated epitopes (HLA I) and 94341 epitopes (HLA II). These epitopes had been derived from human tumor correlation.

### Functional enrichment analysis

The R package enrichplot was employed for the functional enrichment analysis of the genes associated with the circRNAs and the circRNA-derived neoepitopes. These Entrez IDs were then subjected to Kyoto Encyclopedia of Genes and Genomes (KEGG) pathway analysis, which was performed in R using the function enrich KEGG from the package clusterProfiler. When the FDR (Q value) of each pathway did not exceed 20%, the pathway was designated as significantly enriched.

### Statistical analysis and software

The neoepitope pipeline for identifying circRNAs, generating peptides, and predicting HLA class I/II binding affinity (CIRC_neo) is available for download at https://github.com/summerjiaqi/CIRC_neo.git. All statistical analyses were performed in python and R. The p-values less than 0.05 were considered statistically significant.

## Results

### General characteristics of the CIRC-neo pipeline

To investigate the landscape of neoepitopes in human cancer, a simple, performant analytical pipeline for predicting neoepitopes was created in the present study. The pipeline integrates cancer transcriptomics data and was named the CIRC-neo pipeline (**Figure 1A**). CircRNAs may disrupt functional protein domains to cause cancer driver effects. According to the findings, circRNAs could be a source of neoepitopes in cancer cells. Using RNA-seq data, researchers developed a pipeline for identifying circRNA-derived neoepitopes. The circRNAs were identified using ribosomal RNA-depleted total RNA sequencing or poly (A)-depleted RNA sequencing, allowing for more circRNA-derived proteins/peptides to be translated. Therefore, more frameshift peptides were presented on HLA I/II and secreted or released from the cancer cell, to which the immune system could respond (**Supplementary Figure 1A**).

The CIRC-neo workflow had four steps (**Figure 1A**). The first step analyzed raw sequencing data from GBM, BC, and CLL patient samples to identify circRNAs. Since the circRNAs identified using a single method present a high number of false positives, it is recommended that for reliable results, circRNAs that have been predicted using two or more methods should be selected. The present study used two methods for predicting the circRNAs, using find_circ and CIRCexplorer2, respectively. Next, the circRNAs with unique BSJ less than 2 were removed, followed by filtering out the circRNAs with lengths greater than 100 kb. OptiType and HLA-HD were employed to perform HLA genotyping for each sample using the normal tissue RNA-seq and cancer tissue RNA-seq data, respectively. The same results were obtained using default parameters. An overview of the HLA I typing results is provided in **Supplementary Table 1**. In the second step, for each identified circRNA, the nucleotide was translated into the corresponding amino acid. In order to explore whether the circRNAs could also be translated, circRNAs with protein-coding potential were first annotated based on the IRES elements and sequence features. Since circRNAs have a covalently-closed structure, their translation must be based on an unconventional initiation mechanism referred to as the cap-independent translation. The circRNAs were then analyzed for the presence of potential ORFs. Theoretically, the translation could occur in up to three rounds (+0, +1, and +2 frame) if the nucleotides in the circRNAs were not integral multiples of three (**Figure 1B**). In the third step, all neoepitopes were further screened against the reference protein sequences (UniProt protein sequence) and GBM, BC and CLL normal sample protein sequences to filter out those that belonged to the group of naturally occurring peptides in a different protein. The fourth step determined the patient-specific HLA alleles and the HLA-binding affinity ranks of the potential neoepitopes. In HLA class I neoepitopes, false positives were removed again, and the HLA-binding peptides were further consistent with the actual *in vivo* cleavage situation. Netchop3.0 (37) was employed to predict the proteasomal cleavage sites. Peptide-HLA pairs with a binding rank of < 2% were designated as putative neoepitopes in the default setting. Furthermore, the binding peptides from the normal samples were removed, and the most promising candidate HLA binding neoepitopes were screened (**Figure 1A**).

In summary, the proposed CIRC-neo pipeline integrated all steps of the neoepitope identification process. The pipeline applied several layers of filtering to control for false positives and the implementation of a processed and immunologically relevant list of predicted neoepitopes from the circRNAs, which could bind to both class-I and class-II restricted HLA alleles. Every step of the tool selection and filtering strategy was carefully selected in terms of performance and versatility. Indeed, the selection of other bioinformatics tools and filtering strategies is allowed, which renders the process highly individualizable.

### CircRNAs and translation evidence

In order to validate the developed CIRC-neo pipeline, the RNA-seq data of CLL, BC, and GBM were analyzed to identify circRNAs and the circRNA-derived neoepitopes. CIRC-neo was applied to three datasets to demonstrate the identification capabilities of the pipeline. The first dataset comprised the total RNA-seq data originally generated from a bladder cancer study, which analyzed tumor tissues from three patients and matched normal tissues. Bladder cancer is the most common urinary system tumor and ranks ninth among the most common cancers worldwide (38). The second dataset contained total RNA-seq data from CLL samples, which included 2 controls and 13 CLL samples. The third dataset included 12 pairs of circ-RNA-seq from human GBM tissue and paired normal tissue, revealing many circRNAs that were dysregulated in the tumors. GBM is the most common and malignant primary brain tumor, with a poor prognosis (39).

Several translated circRNAs have been identified to date, and these are reported to play key roles in human cancers (20, 22, 40–43). In order to evaluate the neoepitope prediction performance of CIRC-neo, proteins and short peptides encoded by the circRNAs were summarized. For instance, the proteins/peptides generated by circAKT3, circFBXW7, circ-E-Cad, circFNDC3B, and circGprc5a were also detected using CIRC-neo. An integrative analysis was then conducted to predict the potential of all circRNAs in coding for functional peptides. The direct and indirect pieces of evidence in favor of several translated proteins/peptides were integrated to validate the reasonability and credibility of CIRC-neo. In addition, CIRC-neo was applied to predict the interaction between several translated protein peptides and HLA alleles.

GBM is a common and the most malignant primary tumor of the brain with a poor prognosis (39). CircRNA-encoded functional proteins have been described during GBM tumorigenesis (17, 22, 42). Circ-AKT3 evolved from exon 3 to exon 7 of the AKT3 gene in chr1 and has a total length of 524 nucleotides. Previous research has shown that Akt3-174AA, a tumor-suppressor protein encoded by circAKT3, is functional. The tandem “AUG” within the RNA circle in Akt3-174AA may start the translation of a new protein. AKT3-174AA was also found in our dataset, as shown in **Figure 1C**. Akt3-174AA is thought to play a negative regulatory role in regulating the intensity of the PI3K/AKT signal, which is low in GBM tissues (43). This also indicated that circRNAs could be a powerful source of neoepitopes, and the protein sequences encoded by circRNAs could further enrich the antigen library.

Furthermore, the multi-omics evidence from published studies to support the translation of the circFBXW7 is described. The circFBXW7 encodes FBXW7–185AA, which under the mediation role of IRES, regulates the expression of FBXW7 and exerts a tumor suppressor effect in triple-negative breast cancer (TNBC) (44). The FBXW7–185AA protein encoded by circFBXW7 also competes with USP28 to prevent the latter from binding to FBXW7α. Then it promotes the ubiquitination and degradation of C-MYC, which is a key regulator of tumorigenesis in glioma (45). The corresponding protein sequences of FBXW7-185AA were also identified in our data, as depicted in **Figure 1D**. Meanwhile, eight peptides in the FBXW7-185AA protein exhibited a high potential for binding to HLA I, while 21 peptides exhibited the potential of binding to HLA II. These peptides are listed in **Supplementary Table 2**. Therefore, FBXW7–185AA could also be a potential therapeutic target.

ROBO2 encodes a protein that belongs to the ROBO family. Members of the ROBO family are a small subfamily of the immunoglobulin superfamily. The developed CIRC-neo predicted that the second exon of the ROBO2 gene was cyclized and produced a circRNA. The track height on exon 2 is higher in the cancer sample than in the normal sample, as shown in **Supplementary Figure 1B**.

E-cadherin (circ-E-Cad) RNA junction reads in ribosome profiling were detected in the GBM samples, while no junction reads were detected in normal brain samples (41). The study identified a potential IRES that drives a protein that possibly encodes 254 amino acids. The lack of a stop codon in the first-round read caused a frameshift in the second-round translation, generating a circ-E-Cad product with a unique 14-aa tail. In addition, the 14 amino acids containing “TNLCDGGHSHRRGR” were produced in the GBM sample (**Supplementary Figure 1C**). Moreover, “NLCDGGHSHR” was a circRNA-derived neoepitope identified in the GBM dataset.

CircGprc5a was translated into a protein in a cap-independent manner and is, therefore, an example of a protein-coding circRNA in cancer. The circGprc5a encoding “FDTKPMNLCGR” played a biological role in BC (40). Similar to circ-E-Cad, circGprc5a was observed to produce a peptide sequence, though analysis of the produced peptide revealed that it did not bind to the patient’s HLA I/II. The findings revealed that the circCFNDC3B-encoding proteins gave rise to a number of neoepitopes. Previous research has suggested that circFNDC3B may encode a novel protein. The ORF indicated that the putative 218 amino-acid protein required more than one complete circle of circFNDC3B to be translated. CircFNDC3B-218AA could be used as a therapeutic target in the treatment of colon cancer (20).

Furthermore, an internal ribosomal entry site was required for the 5’-cap-independent translation. A few circRNAs comprised the initiation codon and putative ORFs of a favorable length, which suggested an unexpected protein-coding potential of these circRNAs. This finding also indicated that a circRNA encoding a protein was closely associated with the occurrence and inhibition of tumors. This observation suggested that circRNAs could be a powerful source of neoepitopes, and the protein sequences encoded by circRNAs could further enrich the antigen library.

### Identifying the cancer-associated circRNAs

The CIRC-neo pipeline was created to find cancer-specific peptides and evaluate their potential as neoepitopes. The study yielded a comprehensive list of putative circRNA-derived neoepitopes for each sample. CIRC-neo was applied to the RNA-seq data from BC, CLL, and GBM, and the number of circRNAs and circRNA-derived neoepitopes from each sample were counted.

Multi-method consensus approaches were adopted to ensure robust results in the case of suboptimal data. The predicted results obtained using the find_circ and CIRCexplorer2 approaches are presented in **Supplementary Figure 2A**. In order to ensure the reliability of the analysis, CIRC-neo controlled the number of false positives by considering only those circRNAs that were detected using both methods. Overall, the results of this analysis suggested that using a combination of algorithms with possibly different and complementary features could improve detection accuracy.

**Figure 2:**
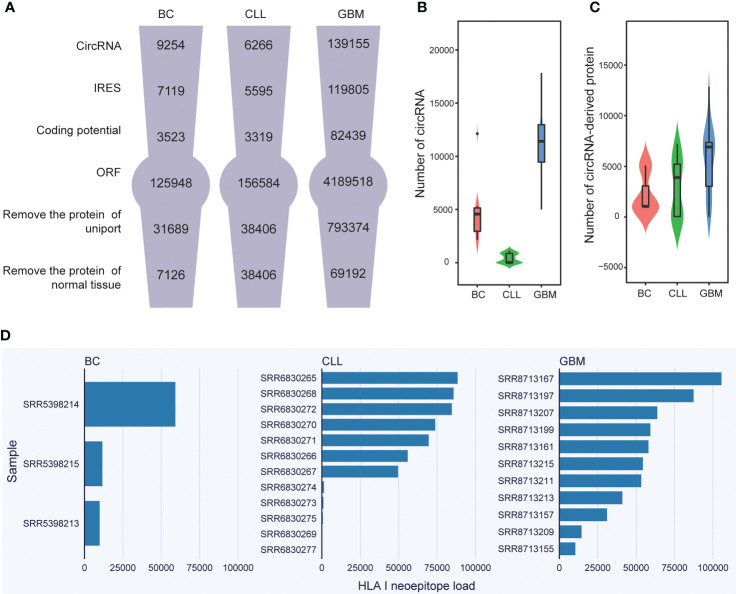
Neoepitope load statistics and analysis. **(A)** Flowcharts presenting the key steps involved in the identification of circRNA-derived neoepitopes. **(B)** The number of circRNAs in BC, CLL, and GBM. **(C)** The cancer-specific circRNA-encoded protein load in BC, CLL, and GBM. **(D)** The HLA class I-binding neoepitope load in BC, CLL, and GBM.

Overall, using the CIRC-neo pipeline developed in the present study, the number of circRNAs in the cancer samples could be determined (**Figure 2A**, first row). In the BC dataset, the number of circRNAs in each sample was 2133, 466 and 2445. In the GBM dataset, the number of circRNAs in 12 tumor samples was 5438, 9296, 11424, 13976, 17835, 14008, 12985, 12722, 6268, 1168, 10404, 13111, respectively. In the CCL dataset, the number of circRNAs in each sample was 984, 739, 636, 993, 2, 832, 914, 1141, 13, 4, 5, 2, 1, respectively. In addition, the results revealed that circRNAs were largely heterogeneous across different types of cancer (**Figure 2B**).

### Identifying the translated circRNAs

Furthermore, a filter was provided to detect the IRES-containing RNA sequences with potential coding capabilities and then predict all possible ORFs for these RNA sequences. Besides considering the AUG initiation protein translation, other initiation codes, such as GUG and UUG, were also considered. Therefore, several ORFs that fulfilled the requirements were identified. However, these circRNA-derived proteins were not cancer-specific. Therefore, the proteins in the protein database (UniProt) and the normal tissues corresponding to them had to be removed (**Figure 2A**). The protein number distribution of each cancer patient in various cancers was then examined. In the case of BC, the three samples contained 1043, 5086, and 997 cancer-specific proteins, respectively. The protein number ranged from 0 to 7,236 in the case of CLL. The fluctuation was greater in the case of GBM, with the maximum number reaching 12,850 and the minimum number being 0. (**Figure 2C**). **Supplementary Table 3** shows the number of potential circRNAs identified from each dataset and the number of candidates obtained from each round of analysis. The distribution of the number of circRNAs in different types of cancer also exhibited wide variability. The samples containing 0 cancer-specific proteins were not analyzed further.

### Identifying the neoepitope load

Importantly, circRNA-derived neoepitopes that could bind to HLA class I or II were identified using the developed CIRC-neo pipeline. In HLA class I neoepitopes, false positives were removed again, and the HLA-binding peptides were further consistent with the actual *in vivo* cleavage situation. The number of peptides obtained by proteasome cleavage is shown in **Supplementary Table 4**. First, the number of such neoepitopes per patient was predicted (**Figure 2D**). The results revealed that the inter-sample variation in the number of circRNA-derived neoepitopes among the different tumor types was extremely high. This higher prevalence of HLA class-II neoepitopes compared to class-I neoepitopes is consistent with the findings of previous studies and also with the greater flexibility of peptide binding in the groove of HLA class-II (46) (**Supplementary Figure 2B**). The neoepitope load varied significantly among the cancer samples, indicating that circRNA-derived neoepitopes could significantly enrich the neoepitope library. Because neoepitopes were not found in normal tissue samples, they could be used as a therapeutic target in cancer treatment.

The IEDB resources were utilized to compile all the recognized human tumor epitopes. The presence of the same epitope would confirm the immunogenicity of the neoepitope predicted in the present study. First, the repeated peptide sequences in IEDB were removed, after which a total of 264,571 HLA class I epitopes and 94,203 HLA class II epitopes remained. If the neoepitope was present in the epitope of IEBD or the epitope of IEDB was present in the dataset of the circRNA-derived neoepitopes, it was assumed that the same peptide existed between the two. The number of identical peptides between the predicted HLA I class neoepitopes and the IEDB tumor-associated epitopes was 51, 305, and 781 in the BC, CLL, and GBM samples, respectively. In addition, whether the predicted HLA II neoepitopes were present in IEDB was determined. It was observed that 685 HLA class II neoepitopes in the BC samples, 10,854 in the CLL samples, and 3465 in the GBM samples overlapped with the corresponding ones in IEDB. While only a few peptides in ten thousand were detected as identical among the large number of circRNA-derived neoepitopes identified in the present study, this result did confirm that circRNA-encoded peptides were potential immunity neoepitopes.

### Features of identified circRNAs and circRNA-derived neoepitopes

**Figure 3:**
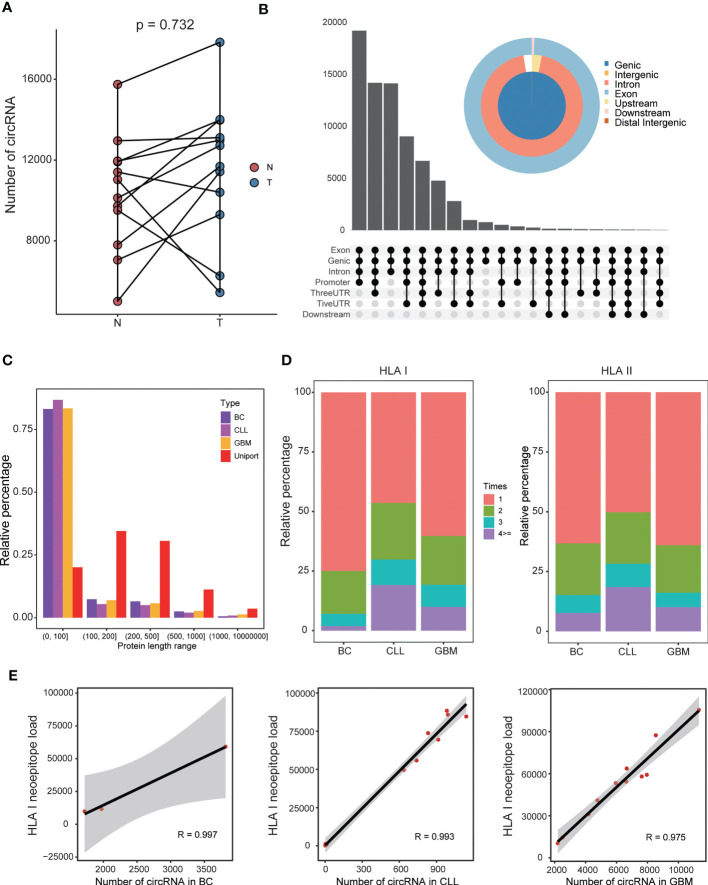
Features of the circRNA-derived neoepitopes discovered in BC, CLL, and GBM. **(A)** The number of circRNAs in GBM and normal tissues. **(B)** The genomic location type of circRNAs in GBM. **(C)** Comparison of the lengths of circRNA-encoded protein and corresponding UniProt protein. **(D)** The proportion of HLA class I peptide and class II peptide appearing 1, 2, 3 and ≥ 4 times, respectively. **(E)** Correlations between the number of circRNAs and the HLA class I-binding neoepitope load.

The CIRC-neo pipeline was applied to analyze the patient samples to demonstrate its utility for identifying neoepitopes and the characteristics of circRNA-derived neoepitopes. First, the quantity characteristic of circRNAs in cancer tissues and normal tissues was explored. The GBM samples had more circRNAs in cancer tissues (p = 0.732), as depicted in **Figure 3A**. Furthermore, the majority of these circRNAs originated from the gene coding region, and nearly all circRNAs contained a portion of the exon region (**Figure 3B** and **Supplementary Figure 3A**, respectively). These findings provided strong support for circRNA translation. The CIRC-neo pipeline was used to discover several circRNA-derived proteins. As depicted in **Figure 3C**, the lengths of these proteins were shorter than those in the UniProt database, and over 75% of the protein sequences contained less than 100 amino acids. The rules for the three datasets were similar, and the protein sequences translated from the circRNAs were shorter in length.

Approximately 25%~50% of HLA class-I neoepitopes and 35%~50% of HLA class-II neoepitopes were shared by more than patient (**Figure 3D**). Overall, the circRNA-derived neoepitopes presented a higher antigen sharing level. Consequently, a subset of these shared circRNAs neoepitopes was expressed in most patients and represented attractive targets or vaccines. Theoretically, the greater the number of circRNAs, the greater the number of circRNA-derived neoepitopes. In the present study, the number of circRNAs was closely associated with the number of HLA class I neoepitopes in all three datasets, and the correlation was greater than 95% (**Figure 3E**). In addition, the number of circRNAs was positively correlated with the number of HLA class II neoepitopes (**Supplementary Figure 3B**).

### Correlation between neoepitope load and immune environment

**Figure 4:**
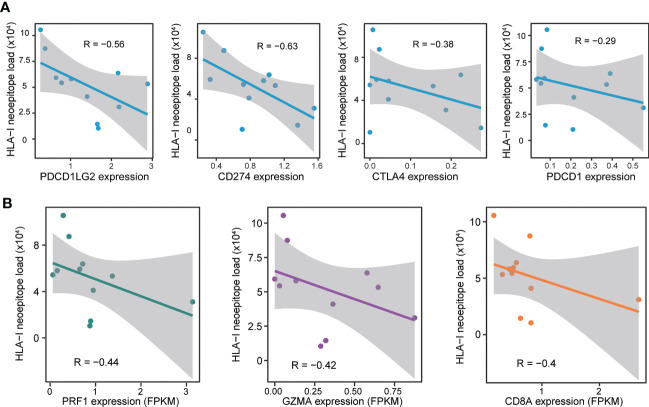
Relationship between the circRNA-derived neoepitope load and immunity. **(A)** Correlation between the neoepitope load and the immune checkpoint-related genes. **(B)** Correlation between the neoepitope load and the markers of immune cytolytic activity. Pearson’s correlation coefficients (denoted as R) are presented in the plots.

The relationship between circRNA-derived neoepitope load and the expression of immune checkpoint-related genes (CD274, CTLA4, PDCD1, and PDCD1LG2) was evaluated to further investigate the correlation between circRNA-derived neoepitope load and the immune environment. The results revealed that in GBM, the number of neoepitopes was negatively correlated with 4 immune checkpoint-related genes (**Figure 4A**), which indicated that certain specific immune checkpoint inhibitors could exhibit greater sensitivity to the GBM patients with a low number of neoepitopes.

Since the circRNA-derived neoepitope load was correlated to the immune checkpoint-related genes, the next step was to examine whether the circRNA-derived neoepitope load was associated with the immune-related genes. The analysis of the relationship between the number of neoepitopes and the immune environment could provide certain suggestions for improving the quality of life of patients. The number of neoepitopes exhibited a negative correlation with the expressions of CD8A, granzyme A (GZMA), and perforin-1 (PRF1), the canonical markers of immune cytolytic activity. Subsequently, the predicted neoepitope load and the immune gene expression were plotted, as depicted in **Figure 4B**.

### Potential functions of identified circRNA-derived neoepitopes

**Figure 5:**
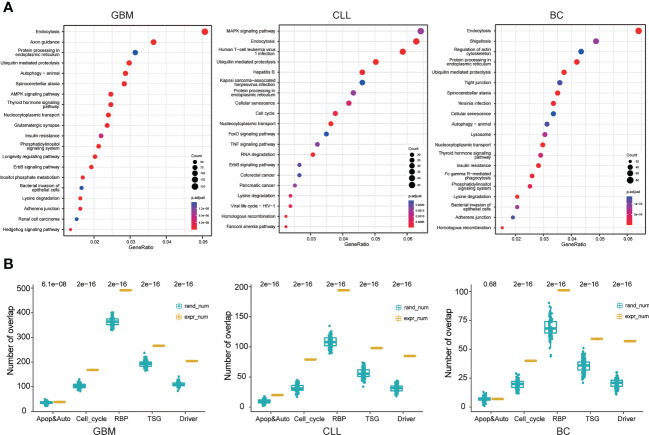
Neoepitope gene function analysis. **(A)** KEGG pathway analysis of the circRNAs in GBM, CLL, and BC patients. **(B)** Functional analysis of circRNA-derived neoepitopes. The yellow bars and blue bars represent the number of circRNA-derived neoepitope genes and the number of randomly selected coding genes, respectively, in apoptosis and autophagy, cell cycle, RNA binding protein (RBP), tumor suppressor gene (TSG), and cancer-related gene sets.

The splicing form of circRNAs contributes to increasing the transcriptomic complexity, which could be exploited in cancer therapy. In this regard, the role of circRNA parent genes and circRNA-derived neoepitope genes in cancer was studied. Next, the circRNA parent genes were subjected to pathway enrichment analysis. The 20 most enriched terms are presented in **Figure 5A**. The three cancer types were generally enriched in endocytosis, ubiquitin-mediated proteolysis, protein processing in the endoplasmic reticulum, nucleocytoplasmic transport, and lysine degradation pathways (p.adj ≤ 0.05). The enrichment results obtained for circRNA parent genes were similar to those obtained for the circRNA-derived neoepitope genes, although the former had a greater number of enriched pathways. Endocytosis is a critical process that allows macromolecules and granular materials from the outside of the cell to enter the cell. Endocytosis is linked to a variety of physiological processes, including immune response, neurotransmitter transport, cell signal transduction, cell and tissue metabolic balance, and so on. Ubiquitin-mediated proteolysis is an important physiological process that keeps cells functioning normally. When the ubiquitin-attached protein moves around the protease, it is recognized and hydrolyzed. Therefore, abnormal ubiquitin-mediated proteolysis may result in human disease. Protein processing, transportation, degradation, and immunity in cells were all linked to ubiquitin-mediated proteolysis. In addition to common pathways, enriched pathways for each cancer type were discovered. The enrichment results obtained for circRNA parent genes were similar to those obtained for the circRNA-derived neoepitope genes, although the former had a greater number of enriched pathways.

The next analysis involved studying the circRNA-derived neoepitope genes in the three cancer datasets, including 3881 genes in the GBM, 1138 genes in the CLL, and 733 genes in the BC. As depicted in **Figure 5B**, a greater number of circRNA-derived neoepitope genes appeared in apoptosis and autophagy, cell cycle, RNA binding protein, tumor suppressor gene, and cancer drive gene sets compared to the randomly selected gene sets. This result indicated that circRNAs or circRNA-encoded peptides could participate in the development and progression of cancer through the modulation of multiple biological processes.

## Discussion

Back-splicing (biogenesis of circRNAs) may alter the ORF of RNA, which would alter the terminal sequences of its protein product. Accumulating evidence suggests that circRNA-encoded proteins play key roles in regulating various cellular events. In addition, the distribution of circRNA is reported to be specific, with the main circRNA distribution observed in the cytoplasm (47). In comparison to somatic mutation, circRNAs have the potential to dramatically alter protein sequences and produce neoepitopes with increased immunogenicity or even specific immunogenicity. The vaccine was reported to be converted into a circular RNA using a series of helper sequences to aid in vaccine circulation translation (48). This further illustrates the superiority of circRNAs translation. Therefore, these circRNAs could be exploited as ideal candidates for immunotherapy. In addition, circRNAs would greatly amplify the source library of neoepitope discovery. In this regard, a robust and thorough tool to identify circRNA-derived neoepitopes was developed in the present study and was named the CIRC-neo pipeline. In addition, a comprehensive and detailed analysis of the identified circRNA-derived neoepitopes was performed.

The CIRC-neo approach was used for identifying the neoepitopes derived from circRNAs. After the identification of circRNAs, their reasonable translation, prediction of HLA and peptide binding, and the integrated automatic analysis screening process led to the identification of the candidate neoepitopes. The CIRC-neo pipeline exhibited certain evident advantages over the other methods available for neoepitope recognition: 1) The translation product prediction based on the RNA-seq data provided a further representative state of the protein repertoire expressed. 2) New sources of neoepitopes were proposed, and the identified novel sources of circRNAs were validated through calculation and confirmed to be reliable. This compensated for the difficulty in distinguishing neoepitopes from non-synonymous mutation sources in cancers with low mutation loads. 3) The interactions between the peptide and HLA II were also studied. 4) Multiple filtering steps were used to eliminate false positives. 5) The parameters could be adjusted based on tumor type or sequencing depth, making CIRC-neo even more suitable for specific research needs.

In order to demonstrate the reliability and effectiveness of CIRC-neo, the circRNAs that have been previously demonstrated to encode peptides/proteins in human cancers were explored and summarized. It was observed that a few of these previously reported peptides/proteins could also be identified using CIRC-neo. In the present study, circRNAs as a source of neoepitopes were identified computationally in cancer samples, and the relationship between the number of neoepitopes and other factors was also determined. In order to investigate the correlation between the predicted neoepitope load and the immune microenvironment, the expression levels of the genes CD8A, GZMA, PRF1, CD274, CTLA4, PDCD1, and PDCD1LG2 were determined. Furthermore, to demonstrate the high sensitivity of the developed CIRC-neo pipeline, the functions of the genes associated with the neoantigens that were closely correlated to protein processing, transportation, degradation, and immunity in cells were determined. The result data supported the hypothesis that circRNAs could be translated into immunogenic peptides, loaded on the HLA, and presented to the immune system. However, detailed integrated molecular characterization of a large cohort of patients is required to identify the features of circRNA-derived neoepitopes.

The identification of truly immunogenic neoepitopes is a difficult process fraught with difficulties. For example, in the current study, the results were analyzed based on an extremely small amount of total RNA or poly (A)-depleted RNA sequencing data, limiting the possibility of drawing a strong conclusion. In-depth analyses on larger patient cohorts, involving both T cell recognition profiling and high-throughput sequencing data, would allow for the impact on immunogenicity and other features to be determined. The identification of circRNA-encoded proteins has been considered a difficult task for a long time, mainly due to the large sequence overlap between circRNAs and the linear mRNAs homologous to host genes. Owing to the unpredictability of intracellular protein degradation and the immune system’s complexity, a major challenge is the rapid and efficient identification of the neoepitopes that are most likely to induce an immune response from high-throughput sequencing data. In addition, personalized RNA or peptide vaccines have been shown to prime host immunity against tumor cells. However, most patients do not experience clinical benefit from these therapies. Improved identification of tumor neoantigens that elicit T cell responses will be needed to increase the scope of benefit from cancer immunotherapy (49). However, the limited TCR sequencing data, the recognition algorithm’s limitations, and the large variety of TCRs affect its application in neoepitope prediction.

The potential circRNA-derived neoepitopes appear promising and are expected to become novel vaccines for cancer treatment in the future. The accurate and comprehensive determination of neoepitope sequences is a critical first step in personalized immunotherapy. This step broadens the researchers’ understanding and provides a convenient and practical tool. The CIRC-neo pipeline proposed here is a computational pipeline for identifying circRNA-derived neoepitopes from RNA-seq data. The prediction of cancer-specific circRNA-derived neoepitopes has the potential to contribute to the development of cancer vaccines.

## Data availability statement

Publicly available datasets were analyzed in this study, these can be found in National Center for Biotechnology Information (https://www.ncbi.nlm.nih.gov/).

## Author contributions

The study conception and design were performed by JX and PZ. Material preparation, data collection, and, analysis were performed by JX, SL and BR. JX was responsible for data analysis and writing of the draft of the manuscript. PZ reviewed/edited the manuscript. All authors contributed to the article and approved the submitted version.
