# Supplementary material for: Circular RNAs as a potential source of neoepitopes in cancer
Source: Front Oncol. 2023 Apr 12;13:1098523. doi: 10.3389/fonc.2023.1098523 (PMC10130363; doi:10.3389/fonc.2023.1098523)
Supplement: Supplementary file 5 [file Image_1.pdf]

Figure S1

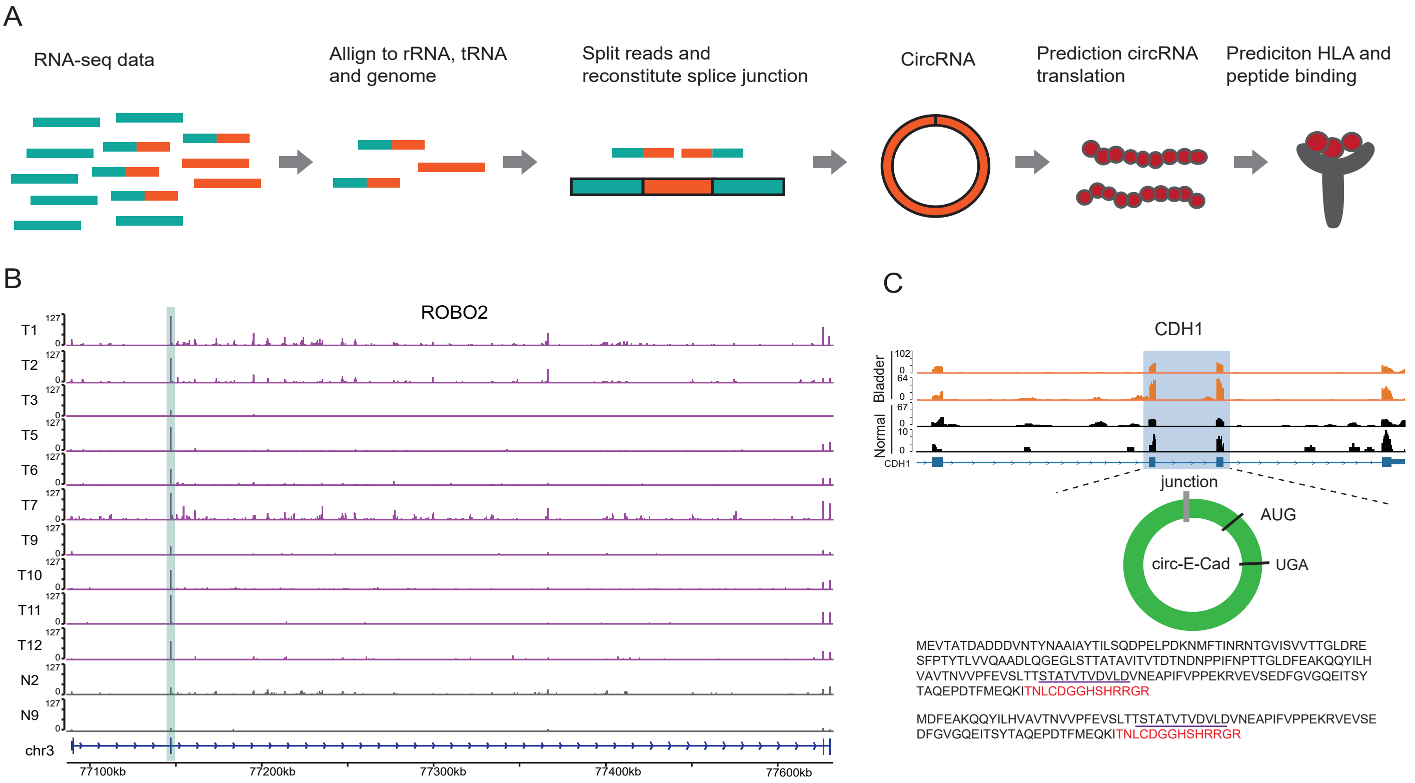

Supplementary Figure 1. The workflow for the identification of neoepitopes using CIRC-neo. **(A)** Flowcharts for the number of circRNA or circRNA-derived neoepitope peptides identified at each stage of the analysis. **(B)** The second exon of ROBO2 forming the circRNA track. Purple represents the GBM samples and gray represents the normal samples. **(C)** The novel proteins and neoepitopes generated from circ-E-Cad. The red letters represent the novel peptide sequences while the purple lines represent HLA class I-binding neoepitopes.
