# Supplementary material for: Circular RNAs as a potential source of neoepitopes in cancer
Source: Front Oncol. 2023 Apr 12;13:1098523. doi: 10.3389/fonc.2023.1098523 (PMC10130363; doi:10.3389/fonc.2023.1098523)
Supplement: Supplementary file 6 [file Image_2.pdf]

Figure S2

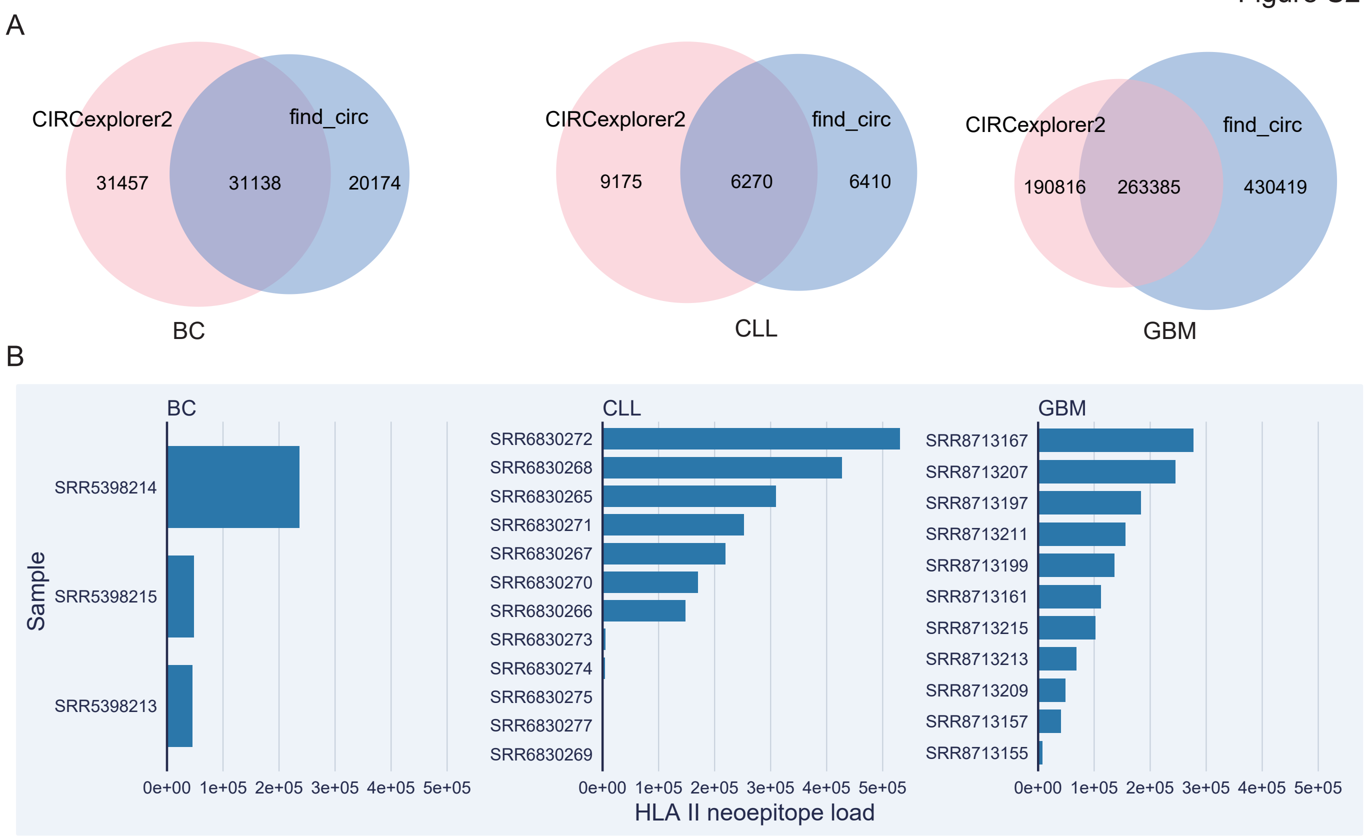

Supplementary Figure 2. The circRNA and HLA class II-binding neoepitopes load in BC, CLL, and GBM. (A) The overlap of the predicted results between find_circ and CIRCexplorer2 in BC, CLL, and GBM. **(B)**. The HLA class II-binding neoepitope load in BC, CLL, and GBM.
