# Supplementary material for: Circular RNAs as a potential source of neoepitopes in cancer
Source: Front Oncol. 2023 Apr 12;13:1098523. doi: 10.3389/fonc.2023.1098523 (PMC10130363; doi:10.3389/fonc.2023.1098523)
Supplement: Supplementary file 7 [file Image_3.pdf]

Figure S3

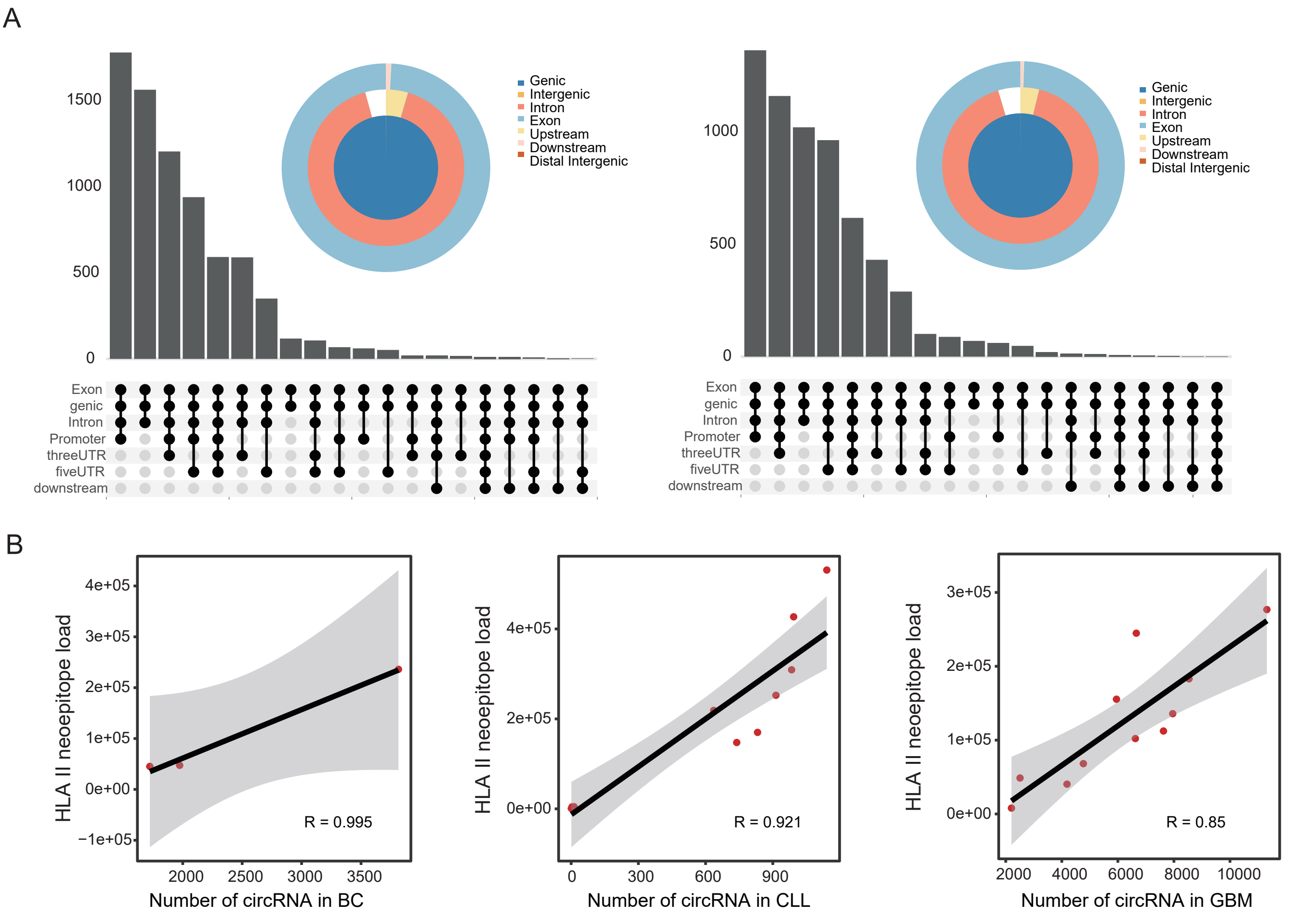

Supplementary Figure 3. The estimates for the circRNA and circRNA-derived neoepitope loads. **(A)** The genomic location type of circRNAs in BC and CLL. **(B)** Correlations between the number of circRNAs and the HLA class II-binding neoepitope load.
